# Proteomic analysis of Biliverdin protected cerebral ischemia–reperfusion injury in rats

**DOI:** 10.1038/s41598-023-47119-3

**Published:** 2023-11-22

**Authors:** Wenya Bai, Siying Huo, Junjie Li, Yuan Yang, Guilin Zhou, Jianlin Shao

**Affiliations:** Department of Anesthesiology, The First Affiliated Hospital of Kunming Medical University, 295 Xichang Road, Kunming, 650032 Yunnan People’s Republic of China

**Keywords:** Computational biology and bioinformatics, Neuroscience

## Abstract

Biliverdin, a heme metabolite, has been previously reported to alleviate cerebral ischemic reperfusion injury (CIRI). However, the alterations of brain proteome profiles underlying this treatment remain elusive. The objective of this study is to analyze the differential protein expression profile in cerebral cortex of rats involved in anti-CIRI effects of Biliverdin, providing experimental foundation for searching specific marker proteins. Rat model of MCAO/R was established, HE staining, TTC staining, TUNEL staining, and neurological behavioral examination, corner turning test, adhesive removal test, were performed to validate the effects of Biliverdin, and the results indicated that Biliverdin plays a significant role in alleviating CIRI. Furthermore, proteomic analysis of brain tissues of rats subjected to CIRI following Biliverdin treatment was performed using an integrated TMT-based quantitative proteomic approach coupled with LC-MS/MS technology to clarify the comprehensive mechanisms of Biliverdin in CIRI. First, we conducted strict quality control data for TMT experiments. Finally, a total of 7366 proteins were identified, of which 95 proteins were differentially expressed (DEPs) between the CIRI group and the Sham group and 52 between the CIRI and BV groups. In addition, two overlapping proteins among the 147 DEPs, Atg4c and Camlg, were validated by RT-qPCR and western blotting, and their levels were consistent with the results of TMT analysis. Taken together, the current findings firstly mapped comprehensive proteomic changes after CIRI treated with Biliverdin, providing a foundation for developing potentially therapeutic targets of anti-CIRI of Biliverdin and clinically prognostic biomarkers of stroke.

## Introduction

Cerebral ischemic stroke is one of the most common clinical diseases, which comprises 84.4% of all stroke types, and is characterized by "high morbidity, high disability rate, high mortality rate, high recurrence rate, and many complications". The disease poses a serious threat to human health^1^. Data from the Global Burden of Disease Research (GBD) show that the number of patients suffering from stroke has been rising steadily since 1990^2^. At present, thrombolytic therapy is the main treatment for stroke, but the therapeutic time window is very short, only 3.5–4 h^3^. Beyond this period, cerebral ischemia–reperfusion injury (CIRI) occurs more readily, which leads to irreversible injury to neurons in the ischemic or reperfusion tissue areas involved. Finally, nerve function deterioration, brain edema, and other serious complications can occur^4^. The high mortality and disability rates of nerve function injury after CIRI seriously reduce the quality of life of patients and place a huge economic burden on families and society. The mechanisms underlying CIRI are complex, and limitations of current medical technology and the clinical prevention and treatment of CIRI still face great challenges. Therefore, study surrounding the mechanisms involved in CIRI is of great significance to correctly understand the pathophysiology of CIRI occurrence and for development of therapeutic treatment and prevention of CIRI.

Biliverdin is a heme metabolite, whose production is catalyzed by heme oxygenase-1 (HO-1). A growing number of studies have indicated the anti-inflammatory, anti-apoptotic, and anti-oxidative stress effects of Biliverdin^5^, which can not only inhibit inflammatory responses induced by Lipopolysaccharide (LPS) but also protect rats from acute lung injury^6^. It can also exert anti-inflammatory effects by inhibiting the expression of Toll-like receptor 4 (TLR4) and the production of inflammatory factors by macrophages^7^. Moreover, other studies have shown that Biliverdin can increase tolerance to ischemia–reperfusion (I/R) in receptor organs by inhibiting nuclear translocation of NF-κB to suppress endogenous NOs production^8^. Our previous study have shown that Biliverdin can suppress the expression of inflammatory factors and alleviate apoptosis in CIRI by modulating the long non-coding RNA H19/microRNA-181b-5p/endothelial cell specific molecule 1 axis^9, 10^. However, the study only clarify the molecular mechanisms by which Biliverdin alleviates CIRI from the micro-RNA and RNA regulatory network, the further mechanism require further elucidation from different aspects.

As the chief executor of life activities of an organism, changes in protein content, structure, and function can systematically and comprehensively reflect bodily responses to environment or disease. The term proteome was first proposed by Wilkins and Williams in 1994, and is a combination of the word protein and genome, principally referring to all proteins in cells or tissues^11, 12^. With the development of detection technology and bioinformatics, the concept of proteomics has emerged with time, and can be employed to analyze protein expression levels, protein post-translational modifications, and protein–protein interactions^13^. Thus, a dynamic and comprehensive analysis of the internal changing processes in the body can be achieved from a holistic perspective. Currently, proteomics has been used mainly to study the underlying mechanisms of pathological changes in diseases or drug intervention mechanisms, and to identify biomarkers of certain diseases^14, 15^.

Proteomics has also been extensively used for studying brain I/R injury^16, 17^. It has been proposed that choledochin participates in the regulation of complex protein networks in the development of CIRI. In this study, a rat model of CIRI was established based on previous studies. Differentially expressed proteins were screened after Biliverdin treatment and the utilization of related technologies in proteomics research. The molecular mechanisms underlying the alleviation of CIRI by Biliverdin lays a theoretical foundation for the clinical treatment of CIRI.

## Material and methods

### Animals

Adult male Sprague–Dawley (SD) rats (specific pathogen-free; 8–12 weeks old; 240 ± 20 g) were obtained from the Experimental Animal Center of Kunming Medical University. All experimental protocols using rats were approved by the Animal Care and Welfare Committee of Kunming Medical University (No. kmmu2021024) and were conducted in accordance with the National Institutes of Health guidelines for the care and use of laboratory animals. Prior to experiments, the rats were housed (ten rats per cage) under constant conditions of 25 ± 2 °C temperature, 50 ± 10% air humidity, and a 12-h light/dark cycle, with ad libitum access to pellet food and tap water.

### Study protocol

After 1 week of acclimatization, the rats were randomly divided into three groups using a random number table: Sham group (Sham group), CIRI group, and Biliverdin treatment group (BV group). Rats in the CIRI and BV groups were operated under isoflurane anesthesia to induce CIRI using a 2 h reversible middle cerebral artery occlusion procedure, followed by 12 h of reperfusion, as previously described, which is referred to as the Middle cerebral artery occlusion–reperfusion (MCAO/R)^18^, and is based on the Stroke Therapy Academic Industry Roundtable (STAIR) recommendations^19^.

### Method of anesthesia

The rats were fasted for 3 h and then anesthetized with isoflurane (Aerrane, Baxter Healthcare Corporation, USA) by inhalation (2.5–3% induction, then 2–2.5% maintenance, with oxygen) at the rate of 1.5 L/min. During the entire procedure, the rats were plated on a thermal blanket to maintain body temperature at 36.5 °C ± 0.5 °C with a regulated heating pad till recovery from anesthesia.

### MCAO/R model establishment

The brief procedure was as follows: After anesthesia, through a middle incision in the neck, the right sides of the common carotid artery (CCA) and external carotid artery (ECA) were exposed and ligated. After the right internal carotid artery (ICA) was isolated, a monofilament nylon suture (Johnson & Johnson, New Brunswick, NJ, USA) was inserted from the CCA to the ICA through a small incision to occlude the middle cerebral artery. The incision was then closed. During the entire procedure, the rats were plated on a thermal blanket to maintain body temperature at 36.5 °C ± 0.5 °C. A laser Doppler system (Peri-Flux System 5000; Perimed, Jarfalla, Sweden) was used to supervise regional cerebral blood flow (rCBF). Rats in which rCBF did not drop < 20% of the baseline levels were excluded from analysis. Rats in the Sham group underwent the same procedure, except for nylon suture insertion. Rats with successful MCAO/R consistently exhibited left-circling behavior.

### Biliverdin administration

Biliverdin (Frontier Scientific, Inc., Logan, UT, USA) was dissolved in NaOH and adjusted to a final pH of 7.4 with HCl. Then, Biliverdin was diluted with 0.9% sodium chloride and stored at 4 °C. Rats in the BV group were intraperitoneally administered 35 mg/kg Biliverdin 15 min prior to reperfusion, then once 4 h after reperfusion, and twice per day thereafter, based on our previous study^20^. In the vehicle control group, the same volume of saline was injected in a similar manner.

### Neurological behavioral examination

Neurological behavioral examination of all rats in the three groups was conducted after 6 h, 12 h, and 24 h reperfusion by an investigator who was blinded to the grouping. The examination was performed according to the 18-point scale system described by Garcia et al.^21^.

### Corner turning test

In order to evaluate coordination function of rats, the corner turning test was performed as previously described, at the timepoints of 7d, 14d after reperfusion. Firstly, rat was left in the test device consisting of two vertical boards at a 30° angle. When entering the corner, the rats will turn left or right. Under normal conditions, rat turned left or right with equal frequency, but the rat suffered from MCAO/R preferentially turned toward the unimpaired (right side in our experiment). This test was repeated 10–15 times, with an interval of more than 30 s. The number of turns in each direction of the rats was observed and recorded. The percentage of turning direction was calculated as: right turns/total turns*100%. A double blind test was adopted.

### Adhesive removal test

The adhesive removal test (ARST) was also performed to evaluate the sensory motor function of rats, at the timepoints of 7d, 14d after reperfusion. Firstly, apply adhesive tape strips on the rats paw. Then observe the rat’s behavior. The contact time (time-to-remove) is defined as the point that the rat reacts to the presence of the adhesive tape strips. For each adhesive, the rat may start by either shaking its paws and/or directly bring its paws to its mouth. Both behaviors indicate that the rat has felt the adhesive tape and represent the end of the time to contact. This test was repeated 3 times, with an interval of more than 6 min. Before conducting the formal ARST, rat were continuously trained to remove adhesive tape strips for 5 days, with 3–5 training sessions per day to reach a ‘plateau’ effect.

### Infarct volume determination

After the neurological deficiency examination, rats with neurological deficiency scores 1, 2, or 3 were sacrificed under deep anesthesia. Then, the brains were removed, cut into 2 mm thick coronal slices, and incubated in 2% 2,3,5-triphenyltetrazolium chloride (TTC, Sigma Aldrich, St. Louis, MO, USA) at 37 °C for 30 min in the dark. After staining, the sections were washed with PBS (3 times, 1 min each) and fixed in 4% paraformaldehyde for 24 h. Color images were acquired using a digital camera (Canon, Japan), and infarct volumes were analyzed using ImageJ 1.4 software (National Institutes of Health, Bethesda, MD, USA). The infarct volumes were normalized to that of the non-occluded hemisphere. This procedure was also performed by another investigator blinded to the grouping.

### Hematoxylin–eosin staining (HE) staining

Brain tissue on the I/R side was fixed with 4% paraformaldehyde, embedded in paraffin, and cut into 4 µm tissue sections. After dehydration and dewaxing, hematoxylin staining was carried out. After differentiation and back blue dehydration, the sections were reimmersed in eosin fine dye solution. After another dehydration, they were sealed with neutral gum and placed under a microscope for observation.

### Terminal deoxynucleotidyl transferase-mediated dUTP-biotin nick end labeling assay (TUNEL) staining

Apoptosis in ischemic brain tissues subjected to various treatments was detected using TUNEL staining. The assay was conducted using the TUNEL Apoptosis assay kit (Servicebio Technology Co. Ltd., Wuhan, China) according to the manufacturer's protocol. Images were captured using a fluorescence microscope. The apoptotic index was calculated as the ratio of TUNEL-positive cells to the total number of apoptotic cells.

### TMT quantitative proteomics research

#### Sample preparation

TMT quantitative mass spectrometry was used to quantify proteins in rats. According to the detector (Novogene Co., Ltd., Beijing, China)) protocol, brain samples obtained from ischemic penumbra were homogenized in liquid nitrogen in grinder, and the pooled powder were lysed with lysis buffer which containing 100 mM NH_4_HCO (pH = 8), 8 M Urea and 0.2% SDS, followed by 5 min of ultrasonication on ice. After centrifugation at 12,000×*g* for 15 min at 4 °C, the supernatants were collected and transferred to new tubes for digestion and subsequent analysis. Protein concentrations were determined using the Bradford protein assay kit, according to the manufacturer’s instructions.

#### Proteolysis

Proteolysis in samples were performed by the method of ultrafiltration-assisted sample preparation (FASP). The methods are as follows:

1. An appropriate amount of DTT was added to 30 μL of protein solution to produce a final concentration of 100 mM. The formulated solution was placed in a bath of boiling water for 5 min and subsequently cooled to room temperature;
2. A total of 200 μL of UA buffer was added, and the mixture was mixed uniformly, transferred into an ultrafiltration centrifuge tube, and 14000*g*, centrifuged for 15 min. This step was repeated to remove the wash buffer, DTT, and other low-molecular-weight components;
3. Then, 100 μL of IAA buffer (100 mM IAA in UA) was added, and the mixture was shaken at 600 rpm for 1 min, reacted at room temperature away from light for 3 min, and 14000*g*, centrifuged for 15 min;
4. A total of 100 μL of UA buffer was added to the mixture, and the mixture was 14000*g*, centrifuged for 15 min. This step was repeated twice;
5. Then, 100 μL of 100 mM TEAB buffer was added to the mixture, which was 14000*g*, centrifuged for 15 min. This step was repeated twice;
6. A total of 40 μL of trypsin buffer (4 μg of trypsin and 40 μL of 100 mM TEAB buffer) was added, and the mixture was shaken at 600 rpm for 1 min and subsequently incubated at 37 °C overnight;
7. The mixture was transferred into a new collection tube and 14000 g centrifuged for 15 min. Then, 40 μL of tenfold diluted 100 mM TEAB buffer was added, and the mixture was 14000*g*, centrifuged for 15 min. The filtrate was collected, and peptide content was estimated by measuring the optical density (OD) at 280 nm with a UV spectrophotometer.

#### TMT labeling

1.5 μg trypsin and CaCl_2_ were added, sample was digested overnight. Formic acid was mixed with digested sample, adjusted pH under 3, and centrifuged at 12,000*g* for 5 min at room temperature. The supernatant was slowly loaded to the C18 desalting column, washed with washing buffer (0. 1% formic acid, 3% acetonitrile) 3 times, then eluted by some elution buffer (0. 1% formic acid, 70% acetonitrile). The eluents of each sample were collected and lyophilized. 100 μl of 0.1 M TEAB buffer was added to reconstitute, and 41 μl of acetonitrile-dissolved 10-plex TMT labeling reagent (Thermo Fisher Scientifc, Odense, Denmark) was added, sample was mixed with shaking for 2 h at room temperature. Then, the reaction was stopped by adding 8% ammonia. All labeling samples were mixed with equal volume, desalted and lyophilized. TMT labeling strategy for samples were shown in Table 1.

**Table 1:** TMT labeling strategy for samples.

Group	Sample	TMT labeling
Sham	1	127N
	2	127C
	3	128N
CIRI	1	128C
	2	129N
	3	129C
BV	1	130N
	2	130C
	3	131

#### Separation of mixed peptides

The TMT-labeled peptides were subsequently fractionated into fractions by high-pH reversed-phase fractionation on the L-3000 HPLC system. Chromatographic conditions: Waters BEH C18 (4.6 × 250 mm, 5 μm), the column oven was set as 45 °C. Mobile phase A (2% acetonitrile, adjusted pH to 10.0 using ammonium hydroxide) and Mobile phase B (98% acetonitrile) were used to develop a gradient elution: 0–10 min, 3–20% solvent B; 10–48 min, 20–40% B; 48–50 min, 40–50% B; 50–53 min, 50–70% B; 53–54 min, 70–100% B, the flow rate was 1 ml/min. The whole process was monitored at the absorbance wavelength of 214 nm, and the fractions were collected at 1 min intervals. Approximate 40 fractions were non-contiguously pooled into final 10 fractions. All fractions were dried under vacuum, and then, reconstituted in 0.1% (v/v) formic acid (FA) in water. The residues were reconstituted in 10 μl of 0.1% formic acid and stored at − 80 °C until LC–MS/MS analysis.

#### LC–MS/MS analysis

EASY-nLCTM 1200 HPLC system (Thermo Fisher Scientifc, Odense, Denmark) coupled with Orbitrap Exploris 480 matched with FAIMS (Thermo Fisher Scientifc, Bremen, Germany), with ion source of Nanospray Flex™ (ESI) were used to collect LC–MS/MS data. In detail, 1 μg of each peptide mixture was directly loaded onto a home-made C18 Nano-Trap column (4.5 cm × 75 μm, 3 μm). A home-made analytical column (25 cm × 150 μm, 1.9 μm) was used and eluted with a two-phase mobile system of mobile phase A (100% water, 0.1% formic acid) and B solution (80% acetonitrile, 0.1% formic acid) with a 90 min linear gradient mode: 0–2 min, 6–15% solvent B; 2–78.5 min, 15–40% B; 78.5–80.5 min, 40–50% B; 80.5–81.5 min, 50–55% B; 81.5–90 min, 55–100% B at a constant flow rate of 600 nL/min. Data-dependent scanning was used for mass spectrometry acquisition, FAIMS compensation voltage was set as − 45 and − 65 respectively, and followed to the same acquisition parameters: full scan range from m/z 350 to 1500 with resolution of 60,000 (at m/z 200), an automatic gain control (AGC) target value was Auto (the optimal capacity was automatically calculated by software calculates according to other parameters) and a maximum ion injection time was Auto. The scan-round time in MS/MS was set to 1 s, and the precursors in the full scan were selected from high to low abundant and fragmented by higher energy collisional dissociation (HCD), where resolution was 30,000 (at m/z 200), the turboTMT + precursor Fit function was turned on, the automatic gain control (AGC) target value was 1 × 10^5^. The maximum ion injection time was Auto, a normalized collision energy was set as 36%, an intensity threshold was 5.0 × 10^3^, and the dynamic exclusion parameter was 45 s.

#### Database search of proteins and data analysis

Raw data were retrieved from the spectrum library separately against the Uniprot database(https://www.uniprot.org/,rattus_norvegicus_uniprot_2020_7_2.fasta (36,160 sequences) using the search engine Proteome Discoverer 2.4 (Thermo Fisher Scientific), and the parameters of search were set as follows: mass tolerance for precursor ion was 10 ppm and mass tolerance for product ion was 0.02 Da. Carbamidomethyl was specified as fixed modifications, Oxidation of methionine (M) and TMT plex were specified as dynamic modification. Acetylation, TMT plex, Met-loss and Met-loss + Acetyl were specified as N-Terminal modification in PD 2.4. A maximum of 2 missed cleavage sites were allowed.

In order to improve the quality of analysis results, the software PD 2.4 further filtered the retrieval results: Peptide Spectrum Matches (PSMs) with a credibility of more than 99% was identified PSMs. The identified protein contains at least 1 unique peptide. The identified PSMs and protein were retained and performed with FDR no more than 1.0%. The protein quantitation results were statistically analyzed by T-test. The proteins whose quantitation significantly different between experimental and control groups, (*p* < 0.05 and FC > 1.2 or FC < 0.833 [fold change, FC]), were defined as differentially expressed proteins (DEPs).

#### Bioinformatic analysis

Gene Ontology (GO) and InterPro (IPR) functional analyses (The Web were http://www.geneontology.org for GO, http://www.ebi.ac.uk/interpro/ FOR IPR) of the differentially expressed proteins (DEPs), including cellular components, biological processes, and molecular functions, were conducted using the interproscan program, and the Kyoto Encyclopedia of Genes and Genomes (KEGG) databases (http://www.genome.jp/kegg/,version: 2/20/17(c)Kanehisa Laboratories) were used to analyze protein families and pathways. Probable protein–protein interactions (PPI) were predicted using the STRING (http://string-db.org/) database, the interaction network of the intersecting target proteins was constructed in Cytoscape software (version 3.7.2), Use the AnalyzeNetwork tool to calculate the network graph, and filter out the top core proteins (display the top 10 core proteins) according to the degree value^22^.

### Real-time quantitative polymerase chain reaction (RT-qPCR)

RT-qPCR was used to determine the mRNA expressions of Atg4c and Camlg. Total RNA was extracted from the ischemic brain tissues using TRIzol reagent (Life Tech:15596026) according to the manufacturer’s protocol. Next, cDNA was synthesized by reverse transcription using a Fastking RT kit (with gDNase). Real-time PCR was performed on a light cycle set with 96 real-time PCR detection system using SYBR Green Master Mix (KAPA KK4601). The following thermocycling conditions were used: denaturation at 95 °C for 10 min, then, 40–45 cycles of 15 s at 95 °C, and 30 s at 60 °C. Finally, mRNA expressions were quantified using the 2^−ΔΔCt^ method and normalized to that of Actin (housekeeping gene). The primer pairs used are listed in Table 2.

**Table 2:** Sequences of primers for RT-qPCR in present study.

Gene	Primer	Sequence
Atg4c	Forward	GAGTATTGTGTTGGTATC
	Reverse	AATGACATCTTCTTAGGA
Camlg	Forward	TTGTCTATATTCGCTCCATT
	Reverse	CACTGTCGTCTTTACCTT
Actin	Forward	TATGGAATCCTGTGGCATC
	Reverse	GTGTTGGCATAGAGGTCTT

### Western blotting

Proteins extracted from hippocampal tissues were electrophoretically separated using 15% SDS-PAGE, and transferred to PVDF membranes (Merck Millipore, Massachusetts, USA), followed by blocking with 5% BSA in TBS-T for 1 h at room temperature. Anti-Camlg (1:1000, Rabbit, Cell Signaling Technology, USA), Anti-Atg4c (1:500, Rabbit, ThermoFisher Scientific, USA), and β‐actin antibodies were added and incubated at 4 °C overnight. Subsequently, the membranes were incubated with an anti-HRP-labeled secondary antibody at room temperature for 1 h. After washing in TBS-T three times for 15 min, the membranes were developed using an ECL western blotting reagent (Biosharp, China) for 1 min before imaging with a gel documentation system (Gel DocTM XR + system, Bio-Rad, California, USA). The mean gray value was measured using ImageJ software, and relative protein expression was normalized to that of β-actin.

### Statistical analysis

The statistical analyses were analyzed by SPSS 26.0 (SPSS Inc., Chicago, IL, USA). and the data are expressed as mean ± standard deviation. The differences between groups were determined by one-way ANOVA followed by Tukey’s multiple-comparison test. A P value less than 0.05 as the threshold was considered statistically significant.

### ARRIVE guidelines statement

I and the co-authors confirm that the study is reported in accordance with ARRIVE guidelines, including the Study design, Sample Size, Randomisation, etc.

### Ethics approval

All experimental protocols using rats were approved by the Animal Care and Welfare Committee of Kunming Medical University (No. kmmu2021024) and were conducted in accordance with the National Institutes of Health guidelines for the care and use of laboratory animals.

## Results

### Biliverdin treatment attenuates CIRI

**Figure 1:**
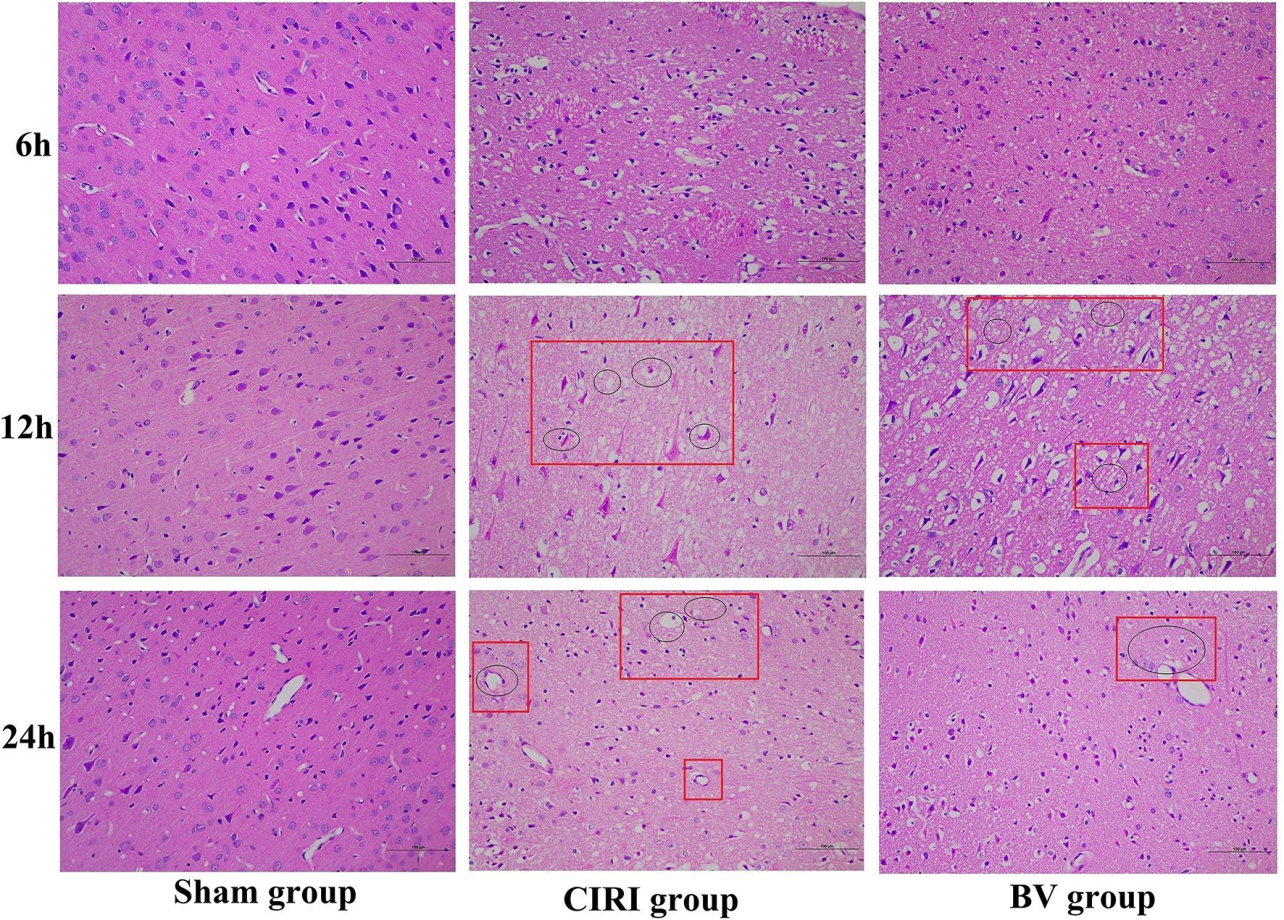
HE staining showing the neuroprotective effect of Biliverdin on cerebral I/R injury. (**A**). HE staining showing the Histological change of rats 6 h, 12 h, 24 h after reperfusion. Scale bar, 100 µm.

**Figure 2:**
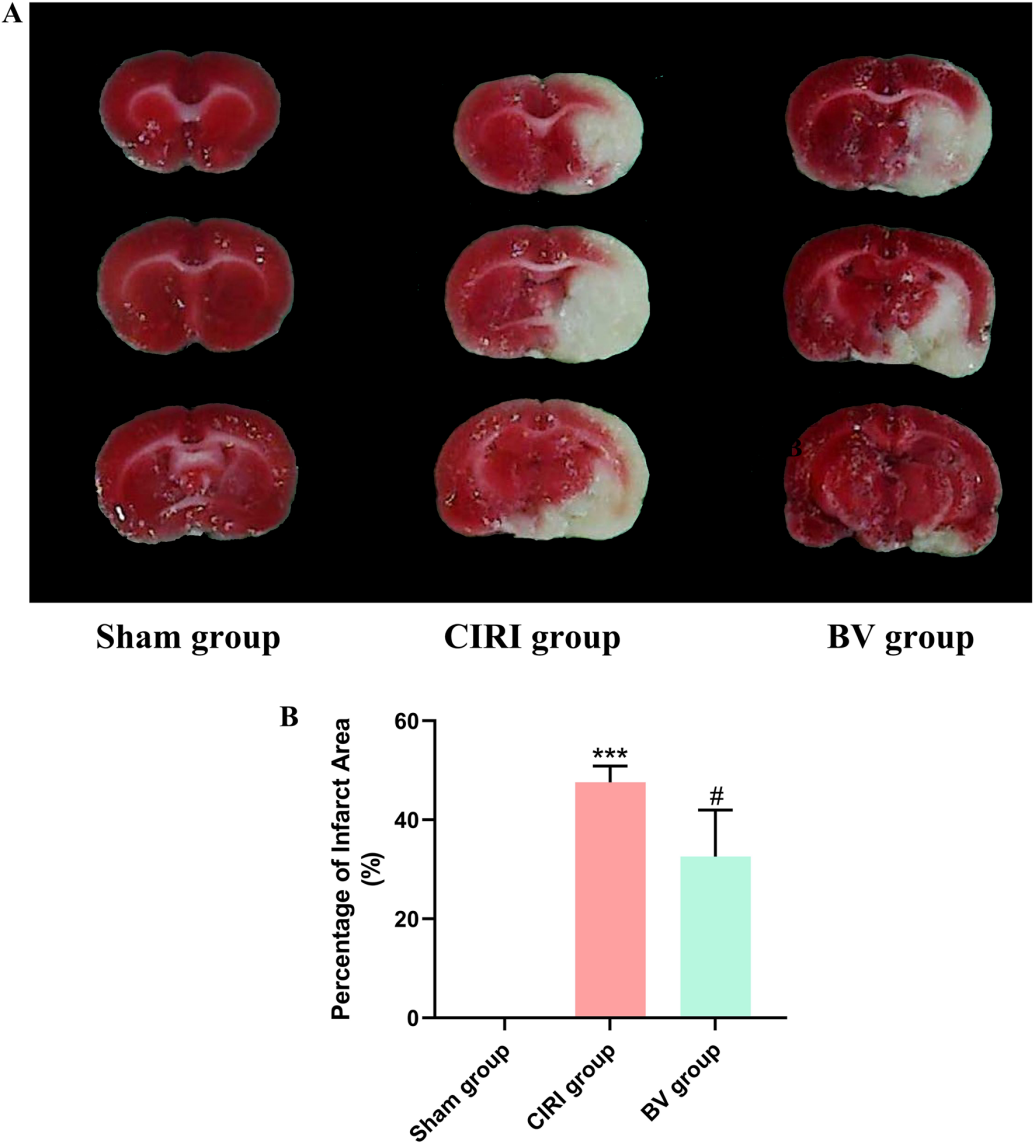
TTC staining showing the neuroprotective effect of Biliverdin on cerebral I/R injury. The infarct volume in rats upon cerebral I/R injury was determined based on TTC staining. *P < 0.05, **P < 0.01, ***P < 0.001 compared to Sham group; ^#^*P* < 0.05, ^##^*P* < 0.01, ^###^*P* < 0.001 compared to CIRI group.

**Figure 3:**
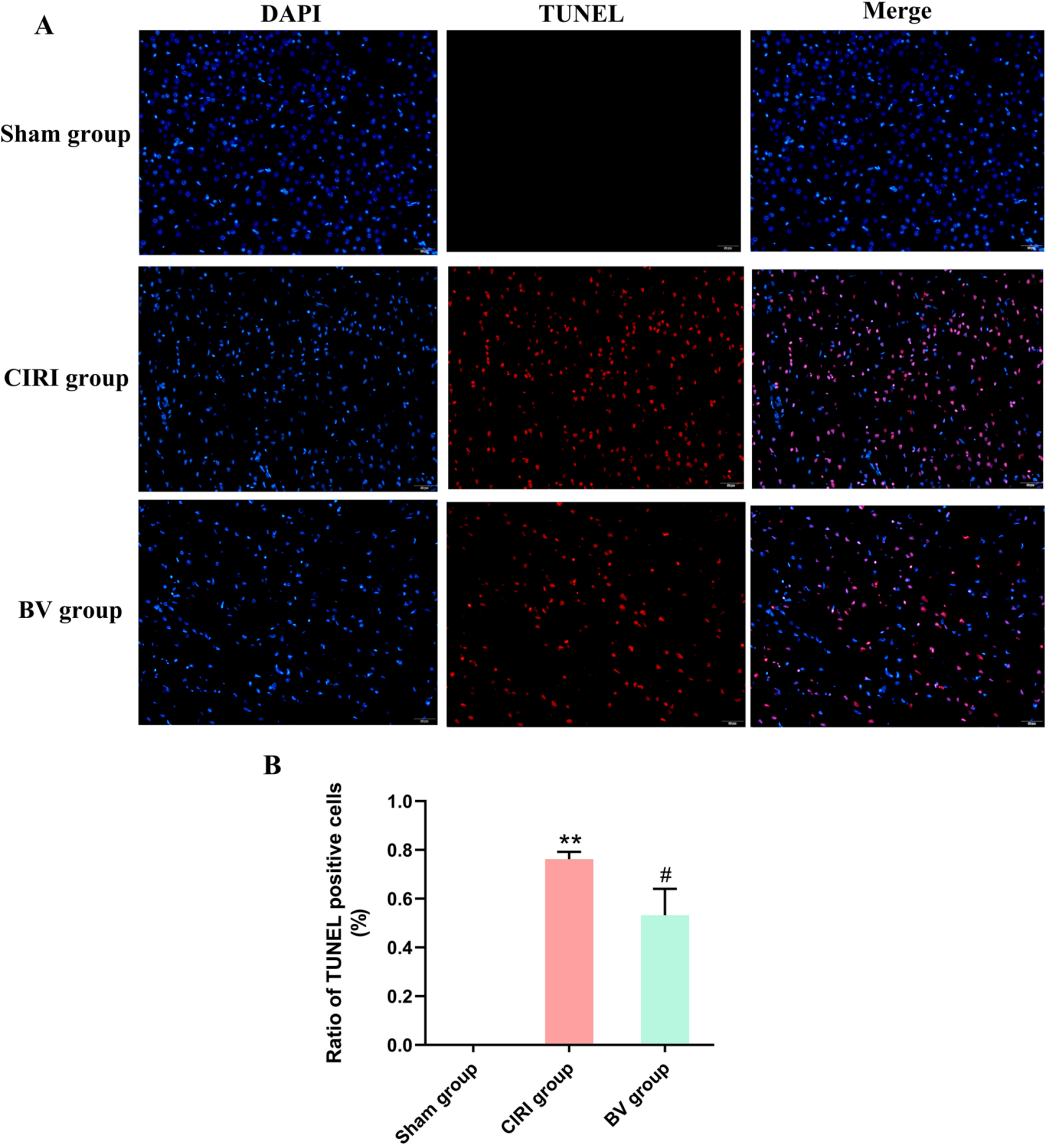
TUNEL staining showing the neuroprotective effect of Biliverdin on cerebral I/R injury. Cerebral cortical neuronal apoptosis was examined by the TUNEL assay (scale bar: 50 μm). *P < 0.05, **P < 0.01, ***P < 0.001 compared to Sham group; ^#^*P* < 0.05, ^##^*P* < 0.01, ^###^*P* < 0.001 compared to CIRI group.

**Figure 4:**
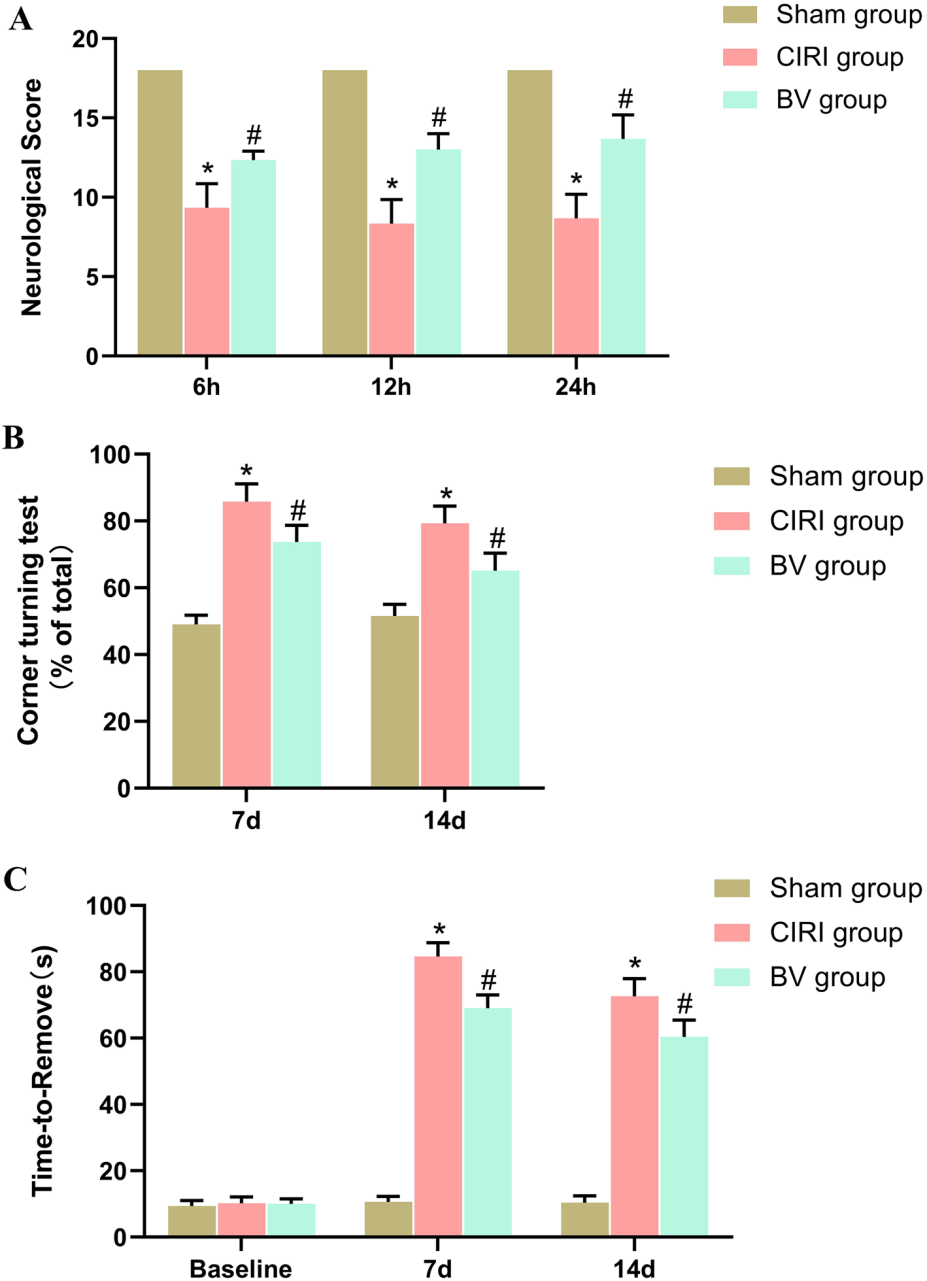
Animal behaviour test showing the neuroprotective effect of Biliverdin on cerebral I/R injury. (**A**) Neurological deficit scores recorded at the timepoints of 6 h, 12 h, 24 h after reperfusion. (**B**) The percentage of turning right in the corner turning test recorded on day 7, 14 after reperfusion; (**C**) The time-to-remove the adhesive tape in the adhesive removal test, recorded on day 7, 14 after reperfusion. *P < 0.05, **P < 0.01, ***P < 0.001 compared to Sham group; ^#^*P* < 0.05, ^##^*P* < 0.01, ^###^*P* < 0.001 compared to CIRI group.

The neuroprotective effects of Biliverdin in CIRI were evaluated based on cell morphology, neurological functions, infarct volumes, and apoptotic cell numbers using HE staining, an 18-point neurologic deficit score, TTC staining, and TUNEL staining. HE-stained sections of the ipsilateral penumbral cortex showed no apparent morphological change in the Sham group, while in the CIRI group, a large number of necrotic vacuoles were formed, and apparent cell shrinkage and enlarged intercellular spaces were observed. However, in the BV group, these phenomena were alleviated, with less neuronal loss (Fig. 1). As shown in Fig. 2, the brain tissues of rats in the Sham group were red with no infraction, while the brain tissues in the CIRI group were stained white, which indicated an apparent infarct. Software analysis results showed that the relative infarct volumes of rats in the CIRI group were greater than those in the Sham group, whereas Biliverdin administration significantly reduced infarct volumes. TUNEL staining for cell death in brain tissue was consistent with these results (Fig. 3). Moreover, our study found that compared to the Sham group, the mNSS score, the percentage of turning right and the time-to-remove the adhesive tape were increased in the CIRI group, and the administration of Biliverdin remarkably improved the neurological deficits, (Fig. 4). Based on these findings, it can be concluded that Biliverdin treatment effectively decreased CIRI.

### Quality control data for TMT experiments

**Figure 5:**
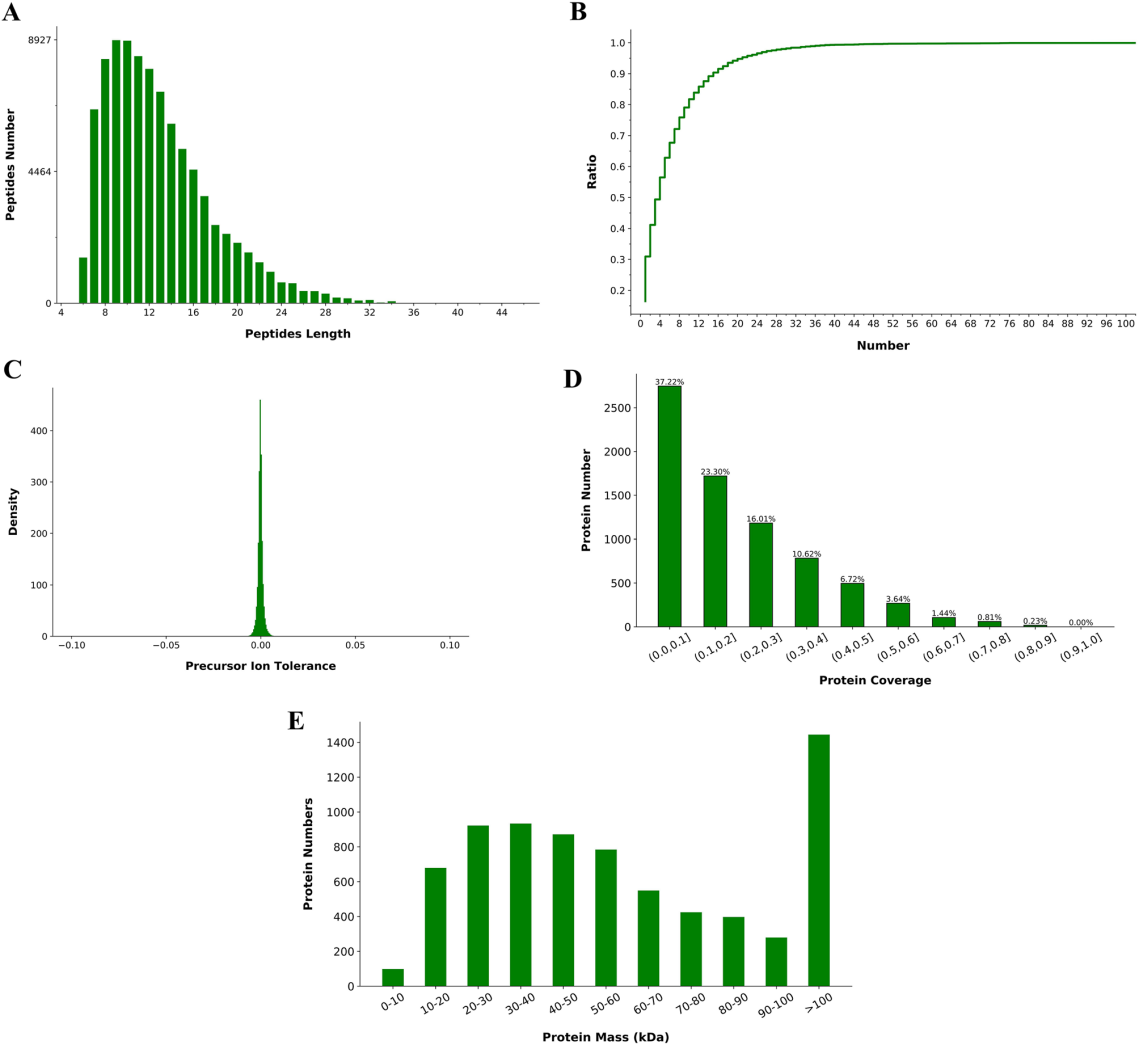
Quality control data for data control. (**A**) The distributions of peptide length. (**B**) Precursor ion tolerance. (**C**) Unique peptide number. (**D**) Protein coverage. (**E**) Protein mass.

**Figure 6:**
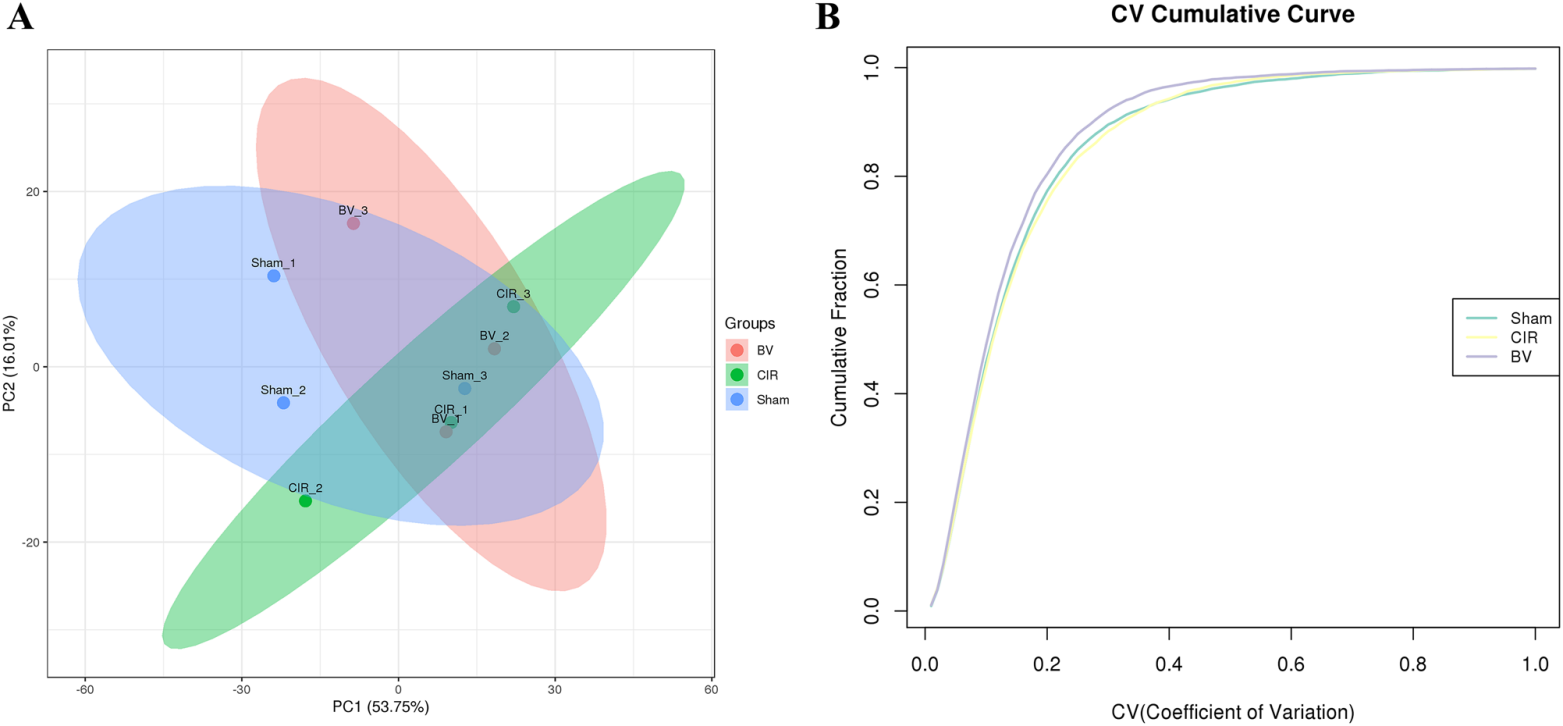
Quality control data for quantitative protein analysis. (**A**) Reproducibility of quantification measured using principal component analysis. (**B**) Reproducibility of quantification measured by coefficient of variation.

First, the quality of the samples was thoroughly verified in terms of protein content and concentration to determine whether they were appropriate for further testing. Second, after the library search for mass spectrometry data was completed, quality control was again performed regarding the distributions of peptides length, precursor ion tolerance, unique peptide number, protein coverage, and protein mass. Finally, the results showed that the identified peptides were mostly between 7 and 25 long, indicating that the protease selected in this study was appropriate (Fig. 5A). The peaks of the precursor ion tolerance distribution mainly concentrated around zero, suggesting that the mass deviation meets the standard requirement (Fig. 5B). The distribution graph of the unique peptide number revealed that as the number increased, the ratio of unique peptide-containing proteins to total proteins slowly increased, indicating that more reliable proteins were identified in this study (Fig. 5C). The quality control of the protein coverage and protein mass distributions showed that the identified proteins are not only highly reliable but also widely distributed (Fig. 5D,E). In addition, during the quantitative protein analysis, principal component analysis (PCA) and protein reproducibility analysis were performed. The PCA results showed that the overall protein differences among the samples in each group and the variability among the samples within the group were small (Fig. 6A), and the cumulative plots of the protein coefficient of variation (CV) in the corresponding samples in each group indicated the high overall reproducibility of the samples (Fig. 6B).

### Identification of DEPs related to Biliverdin administration

**Figure 7:**
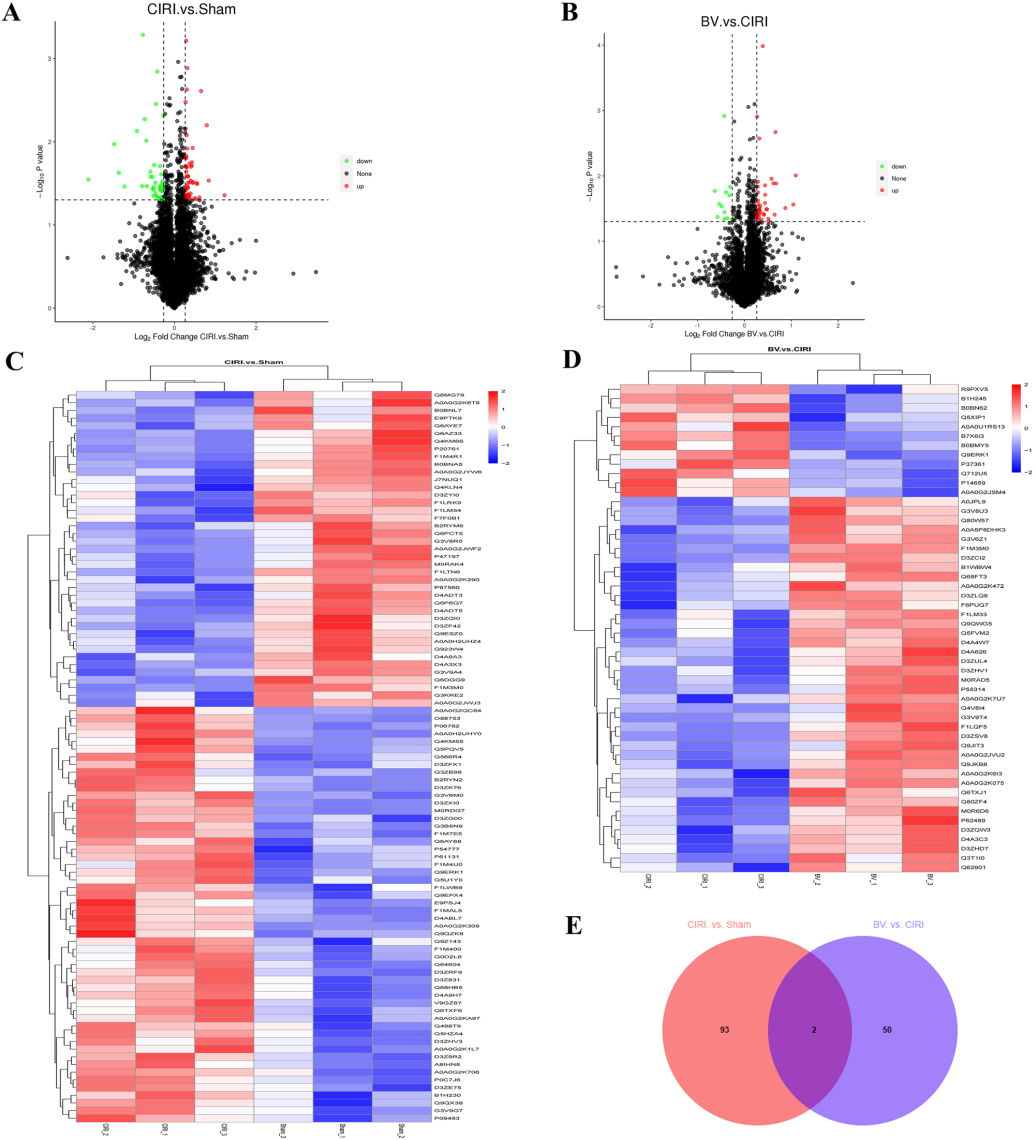
Results of the differentially-expressed proteins (DEPs) identified in the brain tissues. (**A**) The volcano plot illustrating the up- (red) and down-dysregulated (green) DEPs between the CIRI group and the Sham group. (**B**). The volcano plot illustrating the up- (red) and down-dysregulated (green) DEPs between the BV group and the CIRI group. (**C**). The heatmap showing the detail of DEPs between the CIRI group and the Sham group. (**D**). The heatmap showing the detail of DEPs between the BV group and the CIRI group. (**E**). The Venn diagram analysis showing the common dysregulated DEPs between Sham verse CIRI groups and BV verse CIRI groups. The volcano plot was generated using R-3.4.3/ggplot2 software, the heatmap was generated using R-3.4.3/pheatmap, The Venn diagram was drawing on the website: jvenn (inrae.fr).

To better elucidate the molecular mechanisms underlying the neuroprotective action of Biliverdin, a TMT-labelled quantitative proteomic analysis was performed on brain tissues isolated from the three groups employing an HPLC-MS/MS approach in the current study. Three repeated biological tests were conducted for each group, and finally a total of 7366 proteins were identified and quantified with at least one unique peptide matches and false discovery rates (FDRs) < 1% at peptide level. GO and functional annotation were performed. For comparison of different groups, proteins featuring a cut-off with an absolute fold change of < 0.83 (down-regulated proteins) or > 1.2 (up-regulated proteins) and P-value < 0.05 were defined as DEPs. Based on these two rigorous criteria, 95 DEPs were detected in the CIRI group compared with the Sham group, with 54 upregulated and 41 downregulated proteins (Fig. 7A,C). Furthermore, a total of 52 DEPs were detected in the BV group compared with the CIRI group, with 40 upregulated and 12 downregulated proteins (Fig. 7B,D). In order to select the target proteins relating to Biliverdin alleviating CIRI, the study selected DEPs in the CIRI group compared with the Sham group, which is also the DEPs in the BV group compared with the CIRI group. Therefore, Venn diagram analysis were performed and (Atg4c, Camlg) were the overlapping proteins (Fig. 7E), which is consistent with other methods^23, 24^.

### Functional categorization of the differentially expressed proteins

**Figure 8:**
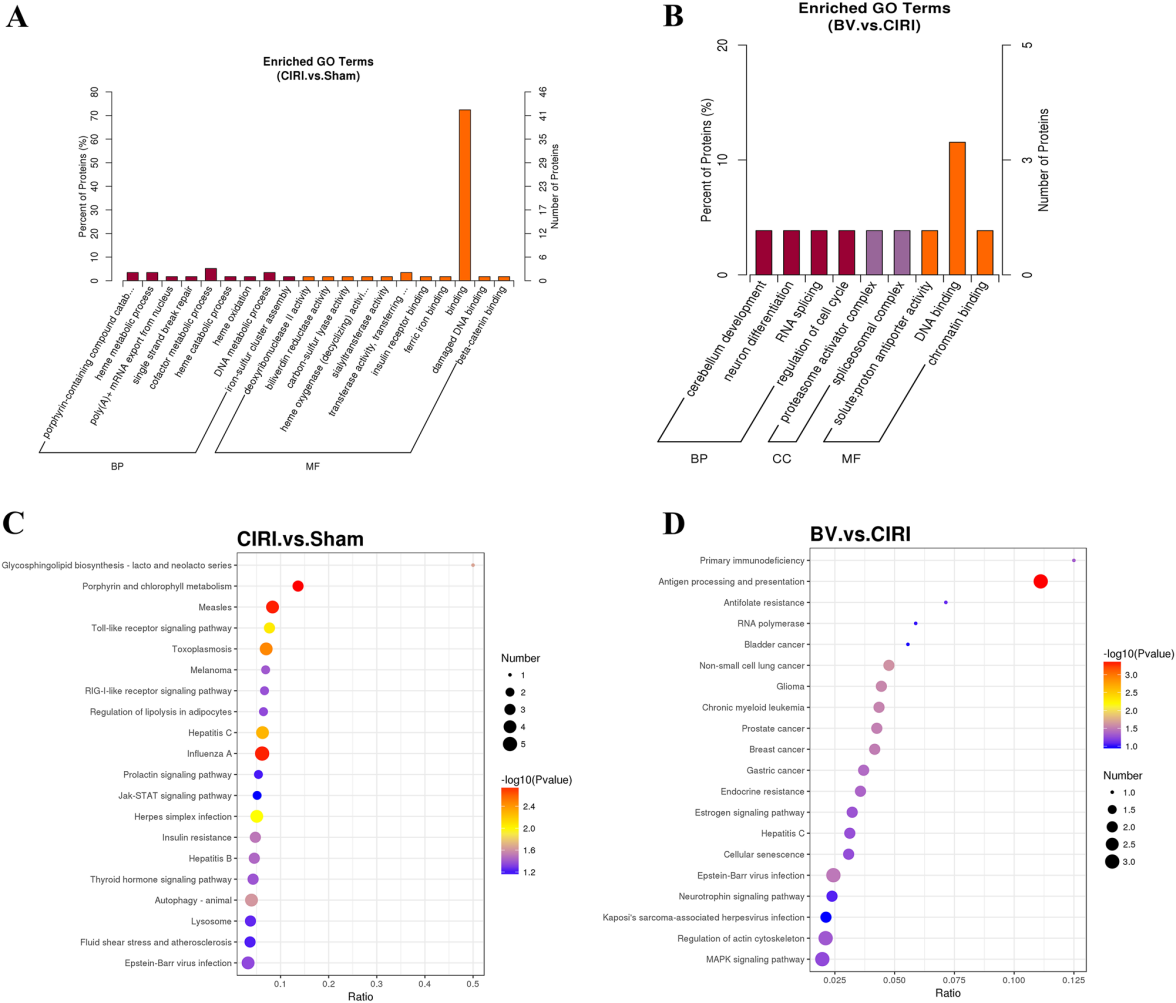
Bioinformatics analysis of DEPs between CIRI verse Sham group and BV verse CIRI group (n = 3 per group). (**A**) Gene ontology enrichment analysis of dysregulated proteins between the CIRI group and Sham group. (**B**) Gene ontology enrichment analysis of dysregulated proteins between the BV group and CIRI group. (**C**) KEGG enrichment analysis of dysregulated proteins between the CIRI group and Sham group. (**D**) KEGG enrichment analysis of dysregulated proteins between the BV group and CIRI group.

To determine potential influences of DEPs and their molecular function following Biliverdin treatment in MCAO/R, GO enrichment analysis and KEGG pathway annotation were conducted. The GO terms included biological process (BP) and molecular function (MF). As shown in Fig. 8A, compared with expression in the Sham group, GO analysis demonstrated that the upregulated DEPs in the CIRI group were significantly associated with heme oxidation, cellular macromolecule biosynthetic process, carbohydrate derivative biosynthetic process, homophilic cell adhesion via plasma membrane adhesion molecules, insulin receptor binding, deoxyribonuclease II activity, and nucleoside diphosphate kinase activity. The downregulated DEPs were significantly associated with cofactor metabolic process, transition metal ion binding, zinc ion binding, DNA binding, and heme catabolic process. In addition, the pathways related to these DEPs included the JAK-STAT signaling pathway, autophagy-animal, TLR-signaling pathway, and RIG-I-like receptor signaling pathway (Fig. 8C).

As shown in Fig. 8B,D, compared with expression in the CIRI group, GO analysis demonstrated that the dysregulated DEPs in the BV group were significantly associated with regulation of cell cycle, neuron differentiation, cerebellum development, thiol-dependent ubiquitin-specific protease activity, and the MAPK signaling pathway. Regulation of actin cytoskeleton, estrogen signaling pathway, antigen processing, and presentation may explain how Biliverdin exerts its neuroprotective effects following CIRI.

### Protein–protein interaction network analysis

**Figure 9:**
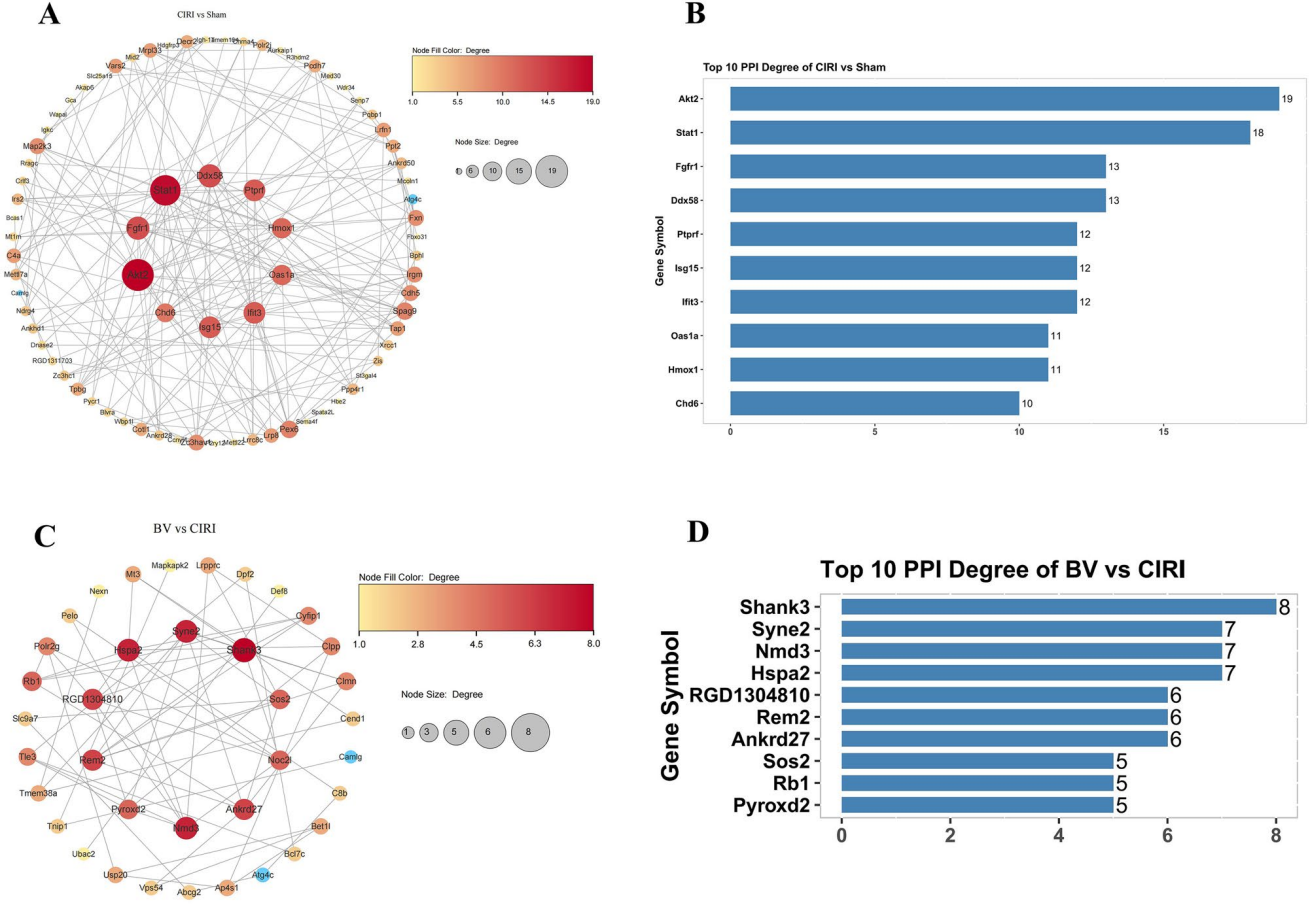
Protein–protein interaction networks of DEPs. (**A**,**B**) PPI network between CIRI and Sham groups. (**C**,**D**) PPI network between BV and CIRI groups. The redder the color and the larger the dot, the greater its Degree value. The lighter the color and the smaller the dot, the smaller the Degree value.

Next, to better comprehend the protein interactions, the STRING database combined with Cytoscape software was used to assess PPI networks of the DEPs in CIRI group vs. Sham group and BV group vs. CIRI group. As shown in Fig. 9A,B, 95 proteins were assigned to the PPI network between the CIRI and Sham groups. The network has a total of 77 nodes and 195 edges, the top 10 core proteins are Akt2, Stat1, Fgfr1, Ddx58, Ptprf, Isg15, Ifit3, Oas1a, Hmox1, Chd6. Furthermore, 52 proteins were assigned to the PPI network between the BV and CIRI groups, the network has a total of 37 nodes and 65 edges, the top 10 core proteins are Shank3, Syne2, Nmd3, Hspa2, GD1304810, Rem2, Ankrd27, Sos2, Rb1, Pyroxd2 (Fig. 9C,D).

### Validation of the potential targets in CIRI rats following Biliverdin administration

**Figure 10:**
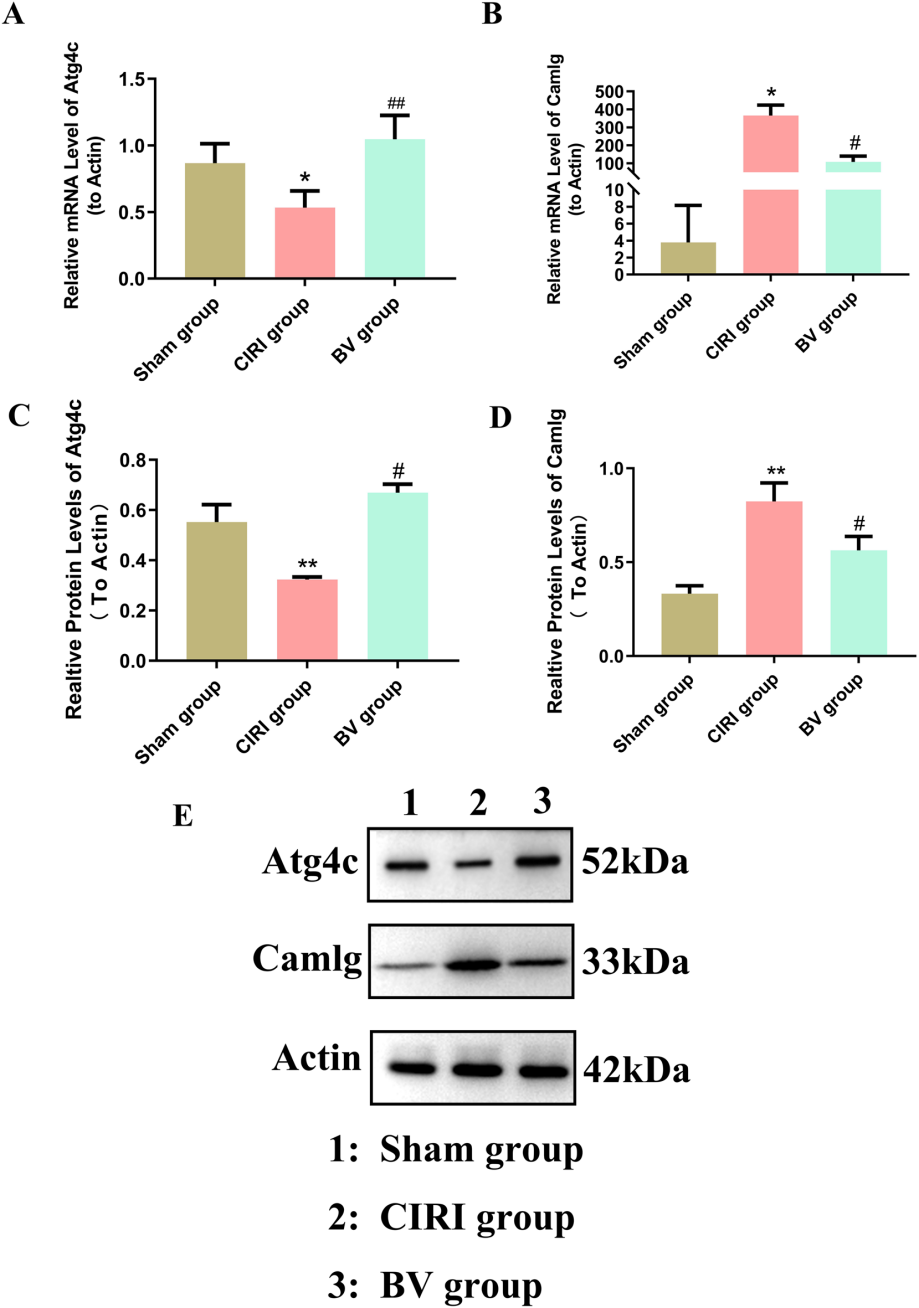
Validation of the overlapping proteins by RT-qPCR and western blotting. (**A**) RT-qPCR assay showing the mRNA expression of Atg4c. (**B**) RT-qPCR assay showing the mRNA expression of Camlg. (**C**–**E**) Western blotting showing the expressions of Atg4c and Camlg. *P < 0.05, **P < 0.01 compared to Sham group; ^#^*P* < 0.05, ^##^*P* < 0.01 compared to CIRI group.

Atg4c and Camlg were chosen for further validation to better understand the molecular mechanisms underlying effect of Biliverdin against CIRI using qPCR and western blotting. The results demonstrated that, compared with the Sham group, the mRNA expression of Atg4c was decreased, while the mRNA expression of Camlg was increased in the CIRI group. However, Biliverdin administration reversed the expression of Atg4c and Camlg (Fig. 10A,B). In addition, the expression of Atg4c detected using Western blot also showed that compared with the Sham group, the expression of Atg4c was decreased, while the expression of Camlg was increased in the CIRI group. However, Biliverdin administration reversed the expression of Atg4c and Camlg, which is consistent with the results of the label-free analysis (Fig. 10C–E, Supplementary Fig. 10E).

## Discussion

Ischemic stroke as well as I/R have attracted worldwide attention, and are responsible for decreasing the quality of life of people worldwide. The pathophysiological process involved in CIRI is a complex, multi-linked enzymatic cascade reaction that involves numerous factors that cause secondary brain injury through various pathways^25^. It has been shown that disturbances in brain energy metabolism^26^, oxidative stress^27^, inflammatory responses^28^, pyroptosis^29^, autophagy^30^, endoplasmic reticulum (ER) stress^31^, and apoptosis^32^. all play important roles in the progression of CIRI. Our previous studies have demonstrated that Biliverdin can alleviate CIRI; however, the mechanism of action of Biliverdin against CIRI has not been fully elucidated. Therefore, further investigations are needed to elucidate potential candidate targets.

To clarify the effects of Biliverdin on CIRI in rats, an MCAO/R model was used to induce CIRI, which has been verified as an effective stroke model to study the pathophysiology of CIRI^33^. The results showed that CIRI resulted in severe behavioral and cognitive abnormalities, whereas Biliverdin treatment remarkably decreased infarct volumes and improved neurological outcomes. Moreover, changes in cell morphology and cell death were also alleviated. Our previous study also shown that Biliverdin can protect against cerebral I/R injury by regulating the miR-27a-3p/Rgs1 axis, thereby inhibiting inflammation and apoptosis^34^, which is consist with the study, but further studies are needed to elucidate the mechanisms underlying the neuroprotective effects of Biliverdin on CIRI.

It is well known that proteins are indispensable for maintaining vital activity, especially when organisms are exposed to endogenous or exogenous beneficial or harmful stimuli. Therefore, proteomic analysis has been utilized to elucidate the molecular mechanisms of drugs used for various diseases. In the current study, TMT-based quantitative proteomics technology coupled with HPLC–MS/MS was applied to comprehensively investigate proteomic changes in penumbral tissue following Biliverdin treatment in rats subjected to cerebral I/R injury. This helped in obtaining a global insight into the molecular networks involved. Our study identified and quantified a total of 7366 proteins in the Sham, CIRI, and BV groups. Among them, 95 DEPs were detected in the CIR group compared with the Sham group, with 54 upregulated and 41 downregulated proteins. A total of 52 DEPs were detected in the BV group compared to the CIRI group, with 40 upregulated and 12 downregulated proteins. Furthermore, GO enrichment analysis and KEGG pathway annotation were performed to identify the potential influence of DEPs and their molecular functions following Biliverdin treatment in MCAO/R. The DEPs in Sham vs. CIRI were primarily involved in heme oxidation, cellular macromolecule biosynthetic process, carbohydrate derivative biosynthetic process, homophilic cell adhesion via plasma membrane adhesion molecules, insulin receptor binding, deoxyribonuclease II activity, nucleoside diphosphate kinase activity, cofactor metabolic process, transition metal ion binding, zinc ion binding, DNA binding, and heme catabolic process, activities possibly being responsible for I/R development and pathogenesis.

The DEPs in BV vs. Sham were primarily involved in the regulation of cell cycle, neuron differentiation, cerebellum development, thiol-dependent ubiquitin-specific protease activity, and the MAPK signaling pathway. Regulation of actin cytoskeleton, estrogen signaling pathway, and antigen processing and presentation may explain how Biliverdin exerts its neuroprotective effects against I/R. The Jak-STAT signaling pathway, autophagy-animal, TLR signaling pathway, and RIG-I-like receptor signaling pathway were the main enrichment pathways related to DEPs.

A Venn diagram analysis revealed that two proteins (Atg4c and Camlg) were co-expressed in these two groups, indicating that these two proteins have a close relationship with the anti-I/R mechanisms underlying Biliverdin’s mode of action. Finally, we validated the two proteins using qPCR and western blotting. The results showed that expression of Atg4c was downregulated and that expression of Camlg was upregulated in the CIRI group; however, BV administration reversed the expression of Atg4c and Camlg, which was consistent with the proteomics results.

Autophagy related 4C (Atg4c), a cysteine peptidase, is a member of the autophagy-related gene family. Atg4c plays a role in the delipidation and deconjugation of LC3 protein, as well as C-terminal peptide cleavage of ATG8, which are essential functions for the assembly of autophagosomes. Therefore, Atg4C is considered to be a critical checkpoint for autophagy regulation^35^. Studies have shown that Atg4C knockdown suppresses glioma progression by inducing cell cycle arrest and promoting apoptosis of glioma cells, possibly by increasing reactive oxygen stress (ROS) production^36^. Moreover, Atg4c expression has certain unique capabilities in Kashin-Beck disease^37^. and epithelial ovarian cancer (EOC)^38^. Liu et al. found that enhancing Atg4c expression protects cardiomyocytes from H/R-stimulated viability loss, apoptosis, and ROS overproduction^39^. Our study identified that expression of Atg4c was downregulated after CIRI, whereas Biliverdin administration could upregulate the expression of Atg4c, suggesting that Biliverdin can inhibit autophagy following CIRI by targeting Atg4c. However, further studies are required to confirm the relationship between Atg4c and CIRI.

Camlg, an ER transmembrane protein, was originally identified as a cyclophilin B-binding protein that regulates Nuclear Factor of Activated T cells (NFAT) -mediated transcriptional activity by elevating cytosolic calcium concentration^40, 41^. Camlg mRNA and protein are highly expressed in many tissues and cell types, including astrocytes, microglia, and neurons^42^. Camlg protein has hydrophilic N- and C-terminal domains and three putative transmembrane domains. The N-terminus of Camlg is integrated into the cell membrane and interacts with other proteins to perform various physiological functions. Junko et al. reported that fibrocystin may participate in the regulation of intracellular Ca^2+^ in distal nephrons by interacting with Camlg^43^, Pinghui et al. found that Kaposi’s sarcoma-associated herpesvirus mitochondrial K7 protein may interact with the N-terminal domain of Camlg to increase intracellular Ca^2+^ levels and block apoptosis^44^. A previous study indicated that Camlg plays important roles in the functional expression and endocytic recycling of postsynaptic γ-aminobutyric acid receptors (GABAARs)^45^. Our study found that the expression of Camlg increased after CIRI; however, Biliverdin decreased the expression of Camlg. We hypothesize that this may be related to changes in intracellular Ca^2+^ concentration. The upregulation of Camlg expression resulted in increased intracellular Ca^2+^ concentrations, which is associated with damage to the mitochondrial membrane and mitochondrial dysfunction, ultimately leading to CIRI. Biliverdin treatment may reduce CIRI by inhibiting Camlg expression.

In addition, PPI network has been constructed to further explore the hub proteins related to the neuroprotective effects of Biliverdin on CIRI. Our findings have demonstrated that Hspa2 is one of the key proteins considered to be related to expression of IL-6, IL-8, IL-15^46^. Many studies have also confirmed that Biliverdin can inhibit the release of pro-inflammatory cytokines following CIRI^9, 34^. Thus, it can be inferred that Hspa2 may be considered as a target for CIRI treatment.

Unfortunately, there are some limitations in the present study. First, the overlapped proteins and the hub proteins from PPI network were not absolutely consistent, because of different research perspectives and algorithms, and we just chose the overlapped proteins to be validated. Perhaps the hub proteins from PPI network need to be validated in the following study.

## Conclusion

In summary, to our knowledge, the comprehensive proteome profiles of brain tissues after CIRI rats treated with Biliverdin were firstly investigated with TMT-based proteomics technique coupled with LC-MS/MS. 95 DEPs were detected in the CIRI group compared with the Sham group, and 52 DEPs were detected in the BV group compared with the CIRI group after statistical analysis and associated with pathophysiological course of CIRI and molecular mechanisms of the anti-CIRI effect of Biliverdin. Finally, Atg4c and Camlg were identified as key proteins, demonstrating that Biliverdin protects against CIRI by regulating autophagy and Ca^2+^ overload. However, the roles of these proteins and the crosstalk between autophagy and Ca^2+^ overload during the procedure of Biliverdin against CIRI are required more investigations. Taken together, the current findings firstly mapped comprehensive proteomic changes after CIRI treated with Biliverdin, providing a foundation for developing potentially therapeutic targets of anti-CIRI of Biliverdin and clinically prognostic biomarkers of stroke.

### Supplementary Information

Supplementary Information 1.

Supplementary Information 2.

Supplementary Information 3.

Supplementary Information 4.

## Data Availability

I and the co-authors confirm that all data generated or analyzed during this study are included in this published article and can also be accessed under reasonable conditions. However, detailed datasets and individual data points are available from the corresponding author on reasonable request. Further, the data in our article is publicly available for the first time, which is generated and analyzed based on our experiment. And our submission above is not associated with any other published article at this time.

## Abbreviations

CIRI: Cerebral ischemic reperfusion injury
DEPs: Differentially expressed
GBD: Global burden of disease
TLR4: Toll-like receptor 4 (TLR4)
MCAO/R: Middle cerebral artery occlusion–reperfusion
CCA: Common carotid artery
ECA: External carotid artery
ICA: Internal carotid artery
BV: Biliverdin
HE: Hematoxylin–eosin
TUNEL: Terminal deoxynucleotidyl transferase-mediated dUTP-biotin nick end labeling assay
RT-qPCR: Real-time quantitative polymerase chain reaction
ER: Endoplasmic reticulum

## References

[1] Lv J (2020). In vivo photoacoustic imaging dynamically monitors the structural and functional changes of ischemic stroke at a very early stage. Theranostics..

[2] Feigin VL (2021). Global, regional, and national burden of stroke and its risk factors, 1990–2019: A systematic analysis for the Global Burden of Disease Study 2019. Lancet Neurol..

[3] Yang P (2020). Endovascular thrombectomy with or without intravenous alteplase in acute stroke. N. Engl. J. Med..

[4] Janus-Laszuk B, Mirowska-Guzel D, Sarzynska-Dlugosz I, Czlonkowska A (2017). Effect of medical complications on the after-stroke rehabilitation outcome. Neurorehabilitation..

[5] Basdeo SA (2016). Suppression of human alloreactive T cells by linear tetrapyrroles; relevance for transplantation. Transl. Res..

[6] Tian WF (2017). Biliverdin protects the isolated rat lungs from ischemia-reperfusion injury via antioxidative, anti-inflammatory and anti-apoptotic effects. Chin. Med. J..

[7] Wegiel B (2011). Biliverdin inhibits Toll-like receptor-4 (TLR4) expression through nitric oxide-dependent nuclear translocation of biliverdin reductase. Proc. Natl. Acad. Sci. USA.

[8] Gibbs PE, Maines MD (2007). Biliverdin inhibits activation of NF-kappaB: Reversal of inhibition by human biliverdin reductase. Int. J. Cancer..

[9] Li JJ (2017). Biliverdin administration ameliorates cerebral ischemia reperfusion injury in rats and is associated with proinflammatory factor downregulation. Exp. Ther. Med..

[10] Li J (2022). Biliverdin modulates the long non-coding RNA H19/microRNA-181b-5p/endothelial cell specific molecule 1 axis to alleviate cerebral ischemia reperfusion injury. Biomed. Pharmacother..

[11] Wasinger VC (1995). Progress with gene-product mapping of the Mollicutes: Mycoplasma genitalium. Electrophoresis..

[12] Wilkins MR (1996). Progress with proteome projects: why all proteins expressed by a genome should be identified and how to do it. Biotechnol. Genet. Eng..

[13] Koehler S (2020). Proteome analysis of isolated podocytes reveals stress responses in glomerular sclerosis. J. Am. Soc. Nephrol..

[14] de Wit M (2014). Colorectal cancer candidate biomarkers identified by tissue secretome proteome profiling. J. Proteomics..

[15] Lao Y, Wang X, Xu N, Zhang H, Xu H (2014). Application of proteomics to determine the mechanism of action of traditional Chinese medicine remedies. J. Ethnopharmacol..

[16] Ma Q (2021). Investigation of brain damage mechanism in middle cerebral artery occlusion/reperfusion rats based on i-TRAQ quantitative proteomics. Exp. Brain Res..

[17] Zhang X (2020). Alterations of brain quantitative proteomics profiling revealed the molecular mechanisms of diosgenin against cerebral ischemia reperfusion effects. J. Proteome Res..

[18] Chiang T, Messing RO, Chou WH (2011). Mouse model of middle cerebral artery occlusion. JOVE J. Vis. Exp..

[19] Recommendations for standards regarding preclinical neuroprotective and restorative drug development. *Stroke*. **30**, 2752 (1999). 10.1161/01.str.30.12.2752 10583007

[20] Zou ZY (2019). Biliverdin administration regulates the microRNA-mRNA expressional network associated with neuroprotection in cerebral ischemia reperfusion injury in rats. Int. J. Mol. Med..

[21] Garcia JH, Wagner S, Liu KF, Hu XJ (1995). Neurological deficit and extent of neuronal necrosis attributable to middle cerebral artery occlusion in rats. Statistical validation. Stroke..

[22] Shannon P (2003). Cytoscape: A software environment for integrated models of biomolecular interaction networks. Genome Res..

[23] Nemethova M (2016). Delayed bradykinin postconditioning modulates intrinsic neuroprotective enzyme expression in the rat CA1 region after cerebral ischemia: A proteomic study. Metab. Brain Dis..

[24] Xu H (2019). Proteomic analysis of hydroxysafflor yellow A against cerebral ischemia/reperfusion injury in rats. Rejuv. Res..

[25] Mandal R (2007). N-hydroxy-pyrroline modification of verapamil exhibits antioxidant protection of the heart against ischemia/reperfusion-induced cardiac dysfunction without compromising its calcium antagonistic activity. J. Pharmacol. Exp. Ther..

[26] Tian WQ, Peng YG, Cui SY, Yao FZ, Li BG (2015). Effects of electroacupuncture of different intensities on energy metabolism of mitochondria of brain cells in rats with cerebral ischemia-reperfusion injury. Chin. J. Integr. Med..

[27] Meng L (2021). TIMP3 attenuates cerebral ischemia/reperfusion-induced apoptosis and oxidative stress in neurocytes by regulating the AKT pathway. Exp. Ther. Med..

[28] Zhang L, Sui R, Zhang L (2022). Fingolimod protects against cerebral ischemia reperfusion injury in rats by reducing inflammatory cytokines and inhibiting the activation of p38 MAPK and NF-kappaB signaling pathways. Neurosci. Lett..

[29] Huang L (2021). Curcumin alleviates cerebral ischemia-reperfusion injury by inhibiting NLRP1-dependent neuronal pyroptosis. Curr. Neurovasc. Res..

[30] Shao ZQ (2021). Apelin-13 inhibits apoptosis and excessive autophagy in cerebral ischemia/reperfusion injury. Neural Regen. Res..

[31] Pan B (2021). Longxuetongluo capsule protects against cerebral ischemia/reperfusion injury through endoplasmic reticulum stress and MAPK-mediated mechanisms. J. Adv. Res..

[32] Wasan H (2022). Dihydromyricetin alleviates cerebral ischemia-reperfusion injury by attenuating apoptosis and astrogliosis in peri-infarct cortex. Neurol. Res..

[33] Ding Y (2014). Neuroprotection by acetyl-11-keto-beta-Boswellic acid, in ischemic brain injury involves the Nrf2/HO-1 defense pathway. Sci. Rep..

[34] Li J (2021). Biliverdin protects against cerebral ischemia/reperfusion injury by regulating the miR-27a-3p/Rgs1 axis. Neuropsych. Dis. Treat..

[35] Sanchez-Wandelmer J, Reggiori F (2017). Atg4 in autophagosome biogenesis. Oncotarget..

[36] Wen ZP (2019). Knockdown ATG4C inhibits gliomas progression and promotes temozolomide chemosensitivity by suppressing autophagic flux. J. Exp. Clin. Cancer Res..

[37] Wu C (2017). Genetic association, mRNA and protein expression analysis identify ATG4C as a susceptibility gene for Kashin-Beck disease. Osteoarthr .Cartil..

[38] Zeng Y, Chen M, Ganesh S, Hu S, Chen H (2020). Clinicopathological and prognostic significance of caveolin-1 and ATG4C expression in the epithelial ovarian cancer. PLoS ONE..

[39] Liu P (2021). Epigallocatechin-3-gallate protects cardiomyocytes from hypoxia-reoxygenation damage via raising autophagy related 4C expression. Bioengineered..

[40] Gladue DP (2018). Classical swine fever virus p7 protein interacts with host protein CAMLG and regulates calcium permeability at the endoplasmic reticulum. Viruses..

[41] Holloway MP, Bram RJ (1998). Co-localization of calcium-modulating cyclophilin ligand with intracellular calcium pools. J. Biol. Chem..

[42] Lee VT, Schneewind O (2001). Protein secretion and the pathogenesis of bacterial infections. Gene Dev..

[43] Nagano J (2005). Fibrocystin interacts with CAML, a protein involved in Ca^2+^ signaling. Biochem. Biophys. Res. Commun..

[44] Feng P (2002). Kaposi's sarcoma-associated herpesvirus mitochondrial K7 protein targets a cellular calcium-modulating cyclophilin ligand to modulate intracellular calcium concentration and inhibit apoptosis. J. Virol..

[45] Yuan X (2008). Calcium-modulating cyclophilin ligand regulates membrane trafficking of postsynaptic GABA(A) receptors. Mol. Cell Neurosci..

[46] Shu W (2020). Triclosan inhibits the activation of human periodontal ligament fibroblasts induced by lipopolysaccharide from *Porphyromonas gingivalis*. J. Biomed. Res..

