# Supplementary material for: Proteomic analysis of Biliverdin protected cerebral ischemia–reperfusion injury in rats
Source: Sci Rep. 2023 Nov 22;13:20525. doi: 10.1038/s41598-023-47119-3 (PMC10665369; doi:10.1038/s41598-023-47119-3)

Figure10E. Original Western blots images of Atg4c for three repeats

Atg4c

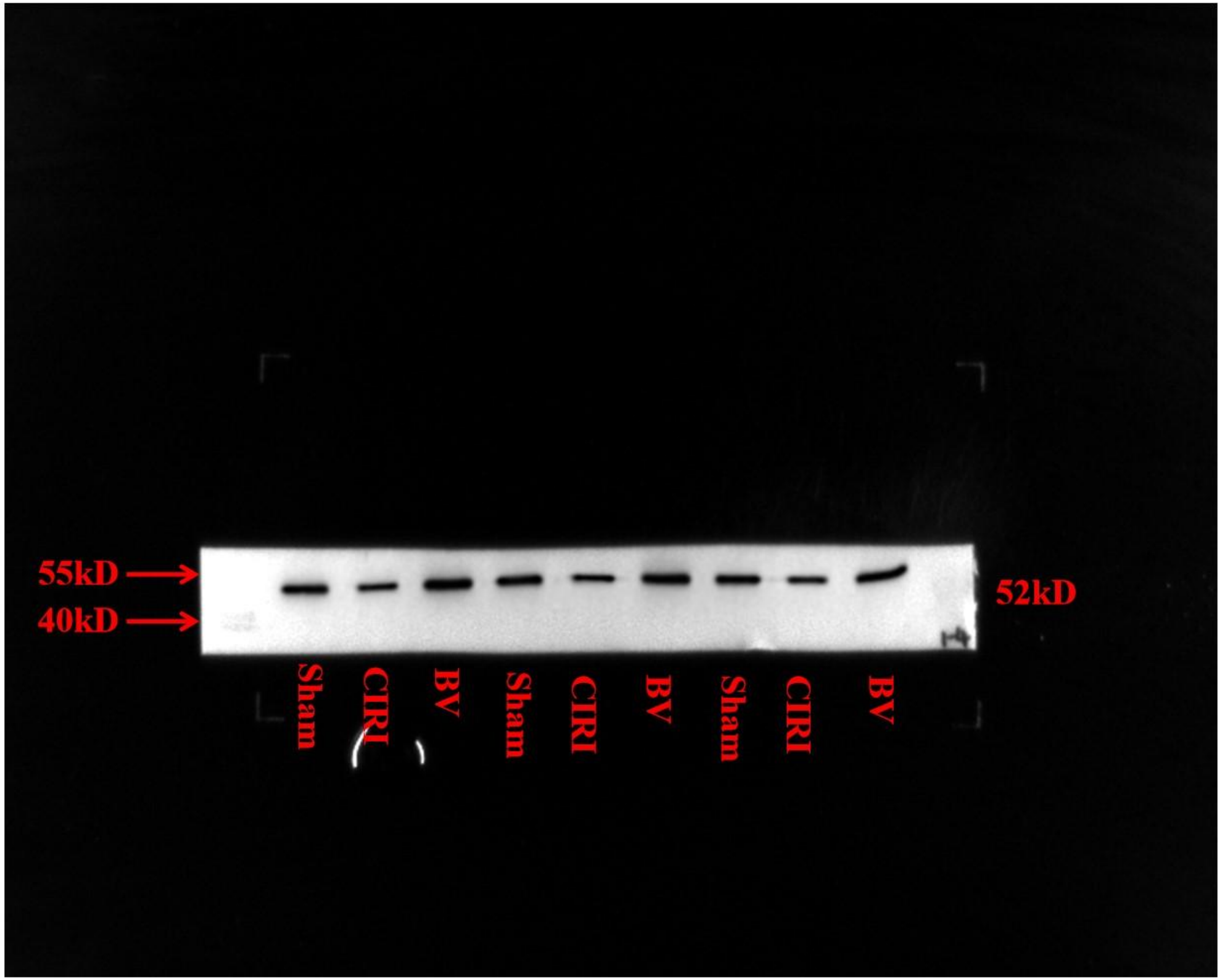

Figure10E. Original Western blots images of Camlg for three repeats

Camlg

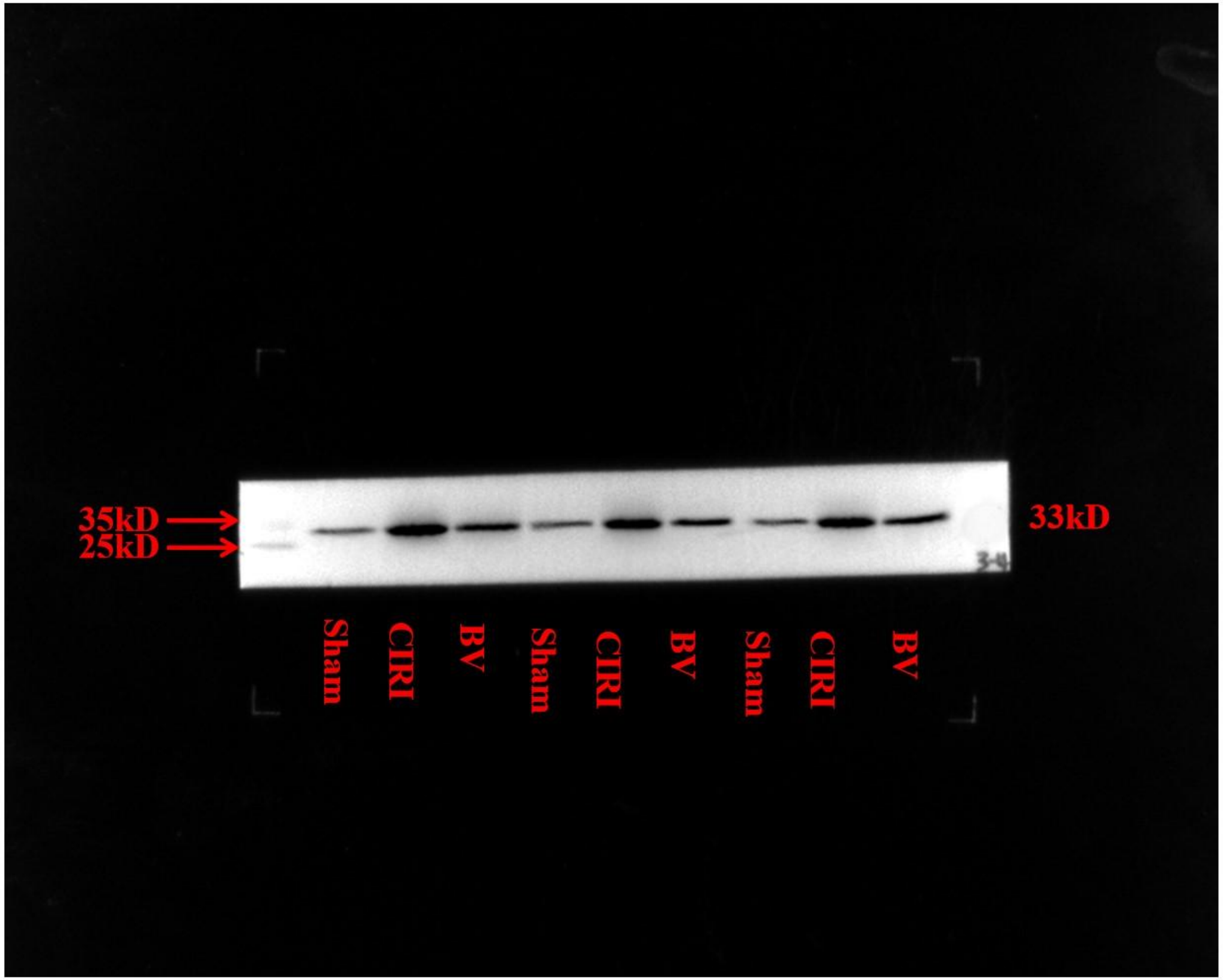

Figure10E. Original Western blots images of β-actin for three repeats

β-actin

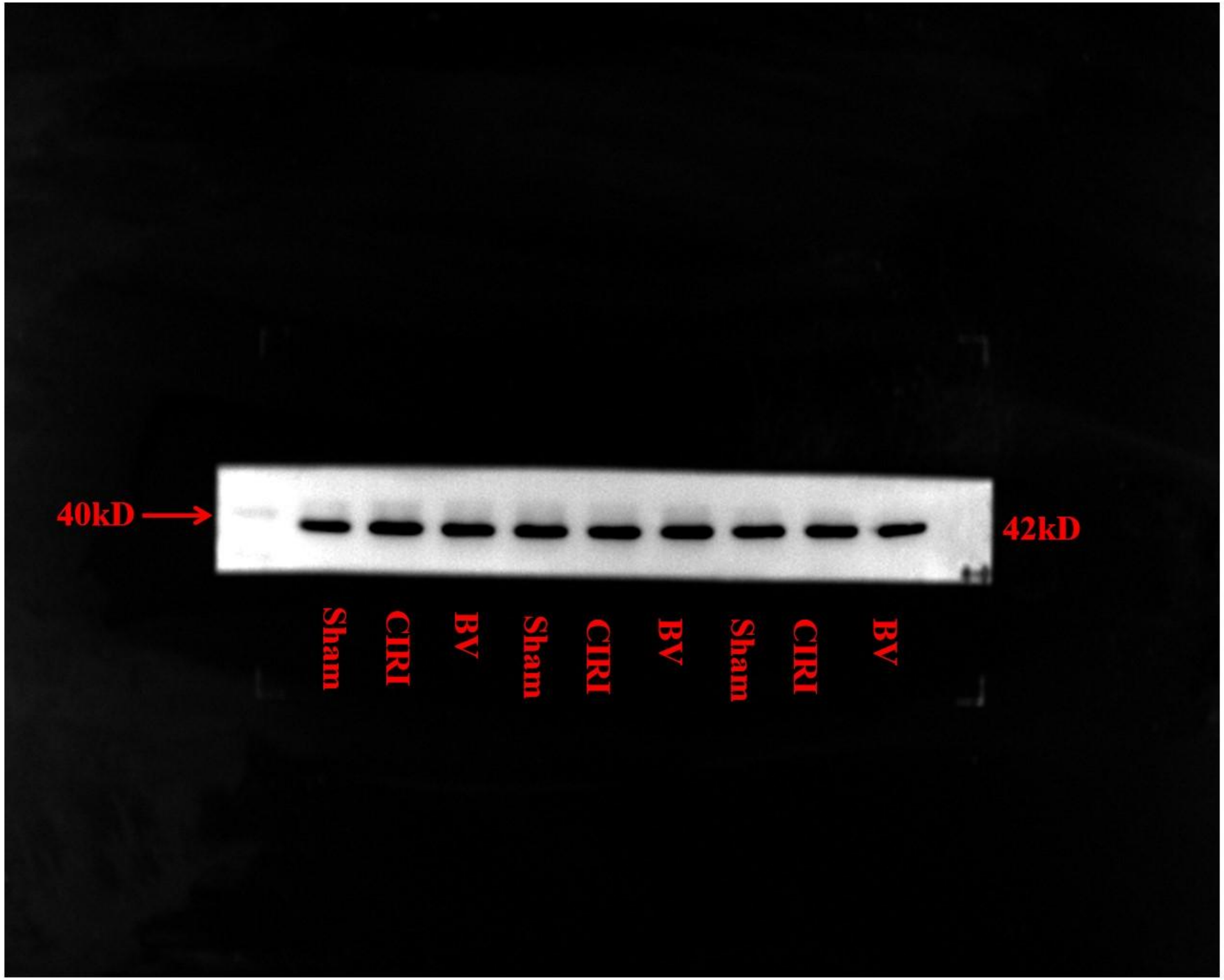

Supplement: Supplementary file 3 — Supplementary Information 3. [file 41598_2023_47119_MOESM3_ESM.pdf]
